# Unique age-related transcriptional signature in the nervous system of the long-lived red sea urchin *Mesocentrotus franciscanus*

**DOI:** 10.1038/s41598-020-66052-3

**Published:** 2020-06-08

**Authors:** Jennifer M. Polinski, Nicholas Kron, Douglas R. Smith, Andrea G. Bodnar

**Affiliations:** 1 Gloucester Marine Genomics Institute, 417 Main Street, Gloucester, MA 01930 USA; 2 Department of Marine Biology and Ecology, Rosenstiel School of Marine and Atmospheric Science, University of Miami, 4600 Rickenbacker Causeway, Miami, FL 33149 United States

**Keywords:** Neuroscience, Neurology

## Abstract

The red sea urchin, *Mesocentrotus franciscanus*, is one the earth’s longest-lived animals, reported to live more than 100 years with indeterminate growth, life-long reproduction and no increase in mortality rate with age. To gain insight into mechanisms associated with longevity and negligible senescence, age-related transcriptional profiles were examined in tissues of the red sea urchin. Genome-wide transcriptional profiling using RNA-Seq revealed few age-related changes in gene expression in muscle and esophagus tissue. In contrast, radial nerve showed an unexpected level of complexity with the expression of 3,370 genes significantly altered more than two-fold with age, including genes involved in nerve function, signaling, metabolism, transcriptional regulation and chromatin modification. There was an age-related upregulation in expression of genes involved in synaptogenesis, axonogenesis and neuroprotection suggesting preservation of neuronal processes with age. There was also an upregulation in expression of positive regulators and key components of the AMPK pathway, autophagy, proteasome function, and the unfolded protein response. This unique age-related gene expression profile in the red sea urchin nervous system may play a role in mitigating the detrimental effects of aging in this long-lived animal.

## Introduction

Gene expression is a key determinant of cellular phenotype, and genome-wide expression analysis can provide insight into the molecular events underlying complex processes such as aging. Large-scale gene expression studies using diverse organisms and tissues are beginning to reveal age-related expression signatures, although the extent to which these represent a predetermined sequence of events that drives aging, or a response to stochastic events (like cellular damage) that accompany aging, is still debated^1,2^.

Age-related gene expression profiles of the short-lived model animals *Caenorhabditis elegans* and *Drosophila melanogaster* share a common adult-onset expression program of genes involved in mitochondrial metabolism, DNA repair, catabolism, peptidolysis and cellular transport^3^. The reduced expression of genes involved in mitochondrial oxidative respiration occurs in early adulthood, before the onset of functional decline, and more abruptly than a damage-response model would predict, which supports the possibility of programmed age-related transcriptional regulation^3^. Gene expression profiles from mice, rats, and humans revealed a common signature of aging in mammals involving overexpression of inflammation and immune response genes and reduced expression of genes associated with mitochondrial function^1,4,5^. Results from mice, monkeys, and humans show an overlap between gene expression and DNA methylation changes during aging and changes occurring during development, which suggest that some aspects of mammalian aging may follow predetermined patterns encoded in the genome as part of the developmental process^6–8^. Analysis of large collections of human tissues indicate a limited number of shared genes associated with age across different tissues, indicating a strong tissue-specific component to aging^9–12^.

Gene expression profiles in animals that exhibit slow aging or negligible senescence may reveal distinct insight into potential mechanisms involved in long-term maintenance of tissues and healthy aging. The naked mole rat (*Heterocephalus glaber*) is a long-lived rodent that can live more than 30 years with sustained reproduction, resistance to common age-related diseases such as cancer, and no age-related increase in mortality rate^13^. Few genes show differential expression with age in tissues (brain, liver and kidney) of naked mole rats and, contrary to other mammals and short-lived model animals, mitochondrial gene expression does not change with age^13^. Gene expression stability during aging was also observed in tissues of the long-lived giant mole-rat (*Fukomys mechowii*) with a maximum lifespan of more than 20 years^14^.

Sea urchins are a well-studied model for developmental biology and an emerging model for the study of longevity, negligible senescence, and tissue regeneration^15–17^. Some species of sea urchin are reported to have long lifespans and exhibit negligible senescence with indeterminate growth, lifelong reproduction, and no increase in size-specific mortality rates^18–20^. The purple sea urchin, *Strongylocentrotus purpuratus*, is reported to live for more than 50 years^21,22^ and, like long-lived rodents, its tissues (muscle, esophagus and nerve) show few age-related changes in gene expression^23^. Based on data from long-lived animals such as the purple sea urchin it has been proposed that lifelong transcriptional stability may be a key determinant of exceptional longevity and negligible senescence^24^.

To further explore this hypothesis, age-related gene expression patterns in tissues of the red sea urchin were investigated. The red sea urchin (*Mesocentrotus franciscanus*) is one of the earth’s longest lived animals, living in excess of 100 years with no age-related increase in mortality rate or decline in reproductive capacity^18–20^. The results revealed a unique age-related gene expression profile in the red sea urchin nervous system that may play a role in mitigating the detrimental effects of aging in this long-lived animal.

## Results

### Age estimates of *M*. *franciscanus*

**Figure 1:**
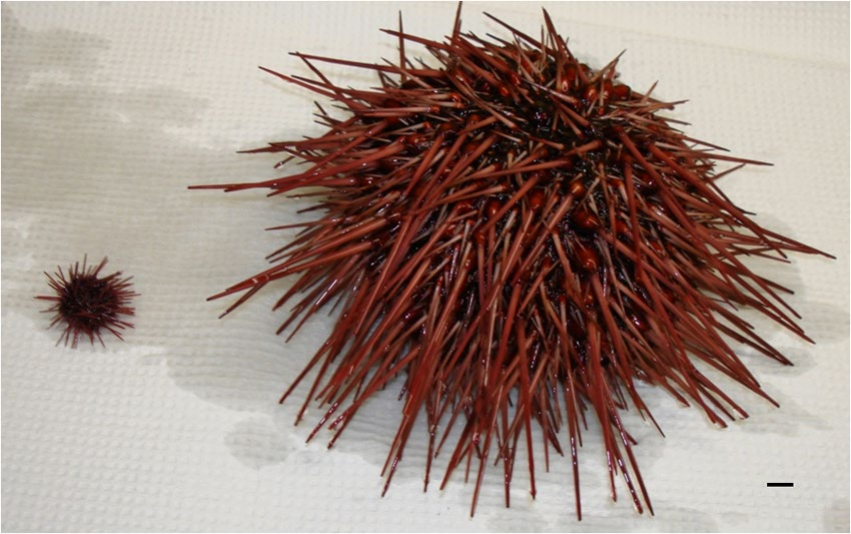
Representative *M. franciscanus* from Small (young) and Large (old) size categories (scale bar = 1 cm).

Collections of *M. fransciscanus* were conducted at the same geographic location and at the same time of year over three years in an attempt to mitigate selecting changes in gene expression that were due to seasonal variation or environmental factors particular to any one year. Ages of individual sea urchins were estimated from test diameter using growth curves generated from the weighted mean of the Tanaka parameters for *M. franciscanus* from tetracycline tagging experiments conducted near our collection site (f = 0.22929, d = 6.07531, a = 0.19906)^25,26^ (Table 1). The Tanaka growth model was previously found to be the best fit for data from sea urchins which exhibit sigmoid growth without an apparent asymptote^25^. As growth rate can be affected by environmental factors (e.g. food availability) the age estimates serve only as a guideline and not absolute values. However, studying animals within a defined study site should give a reasonable indication of relative age within the population. Individual sea urchins were selected from the smallest (<5 cm test diameter) and largest (>15 cm test diameter) size categories each year to ensure a good age separation between the small/young and large/old groups (Fig. 1).

**Table 1:** Estimated ages of small and large *M. franciscanus* used for RNA-Seq.

Sample	Test Diameter (mm)	Est. Age^a^ (years)	Number of animals (n)
Mf – Small (2010)	42.3 ± 1.0	3.5 ± 0.1	6
Mf – Large (2010)	162.2 ± 1.0	144.4 ± 6.6	6
Mf – Small (2011)	48.2 ± 4.1	3.8 ± 0.2	6
Mf – Large (2011)	159.5 ± 1.3	128.1 ± 7.4	6
Mf – Small (2013)	42.2 ± 1.7	3.5 ± 0.1	6
Mf – Large (2013)	157.0 ± 1.5	113.8 ± 7.8	4

^a^Age estimates were calculated as described by Ebert *et al*.^25^ and Ebert, 1998^26^. The values are expressed as mean ± standard error.

### RNA Sequencing and age-related differential gene expression in *M. franciscanus* tissues

RNA from Aristotle’s lantern muscle (ALM), esophagus (ES), and radial nerve cord (RN) from sea urchins of each size category (Small and Large) for the collections conducted over three different summers were used for RNA-Seq analysis (Table 1). RNA was pooled from individuals for each tissue type and size category (ALM-S, ALM-L, ES-S, ES-L, RN-S and RN-L) and samples from each of the three sampling years were treated as independent replicates resulting in a total 18 samples. Sequencing resulted in 255,054,871 read pairs, of which 204,820,674 remained after quality filtering. Trinity assembly resulted in 233,762 transcript contigs (N50 = 1,919 bp), which were clustered into 127,198 “gene” groupings. The transcripts included a total of 268,562,424 bases with an average contig length of 1148 bp, a minimum length of 278 bp, and a maximum length of 15,725 bp. Transcriptome statistics and length distribution of the master transcriptome are shown in Supplementary Fig. S1. BUSCO analysis suggested a near-complete transcriptome with 96.4% of the 978 BUSCO genes found complete as single copies (24.8%) or duplicated (71.6%), and 3% present but fragmented. Functional annotation of the assembled transcript contigs was performed by comparison against sequences of *S. purpuratus* (SPU genes, peptides, and transcriptome from www.EchinoBase.org). This resulted in 44,088 of the 127,198 transcripts matching to 17,292 SPU proteins with top hits averaging 77.7% identity. Gene names used to refer to *M. franciscanus* genes in the text correspond to the gene names of *S. purpuratus* homologs from EchinoBase, omitting the “Sp-“ prefix.

Count-based differential gene expression between Small (young) and Large (old) samples was analyzed within each tissue type and averaged over all three years to yield 144, 123 and 3,370 genes that were significantly different in ALM, ES and RN, respectively [q-value (FDR) < 0.05, log2 fold-change < −1 or>1].

**Figure 2:**
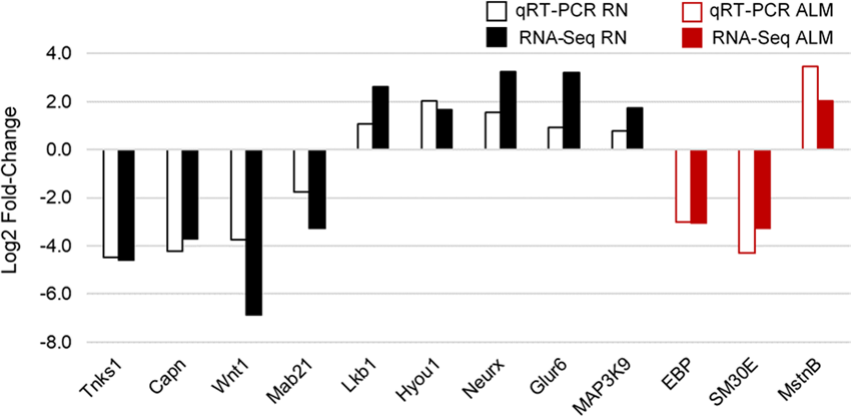
qRT-PCR validation of expression of selected genes in radial nerve cord (RN) and Aristotle’s lantern muscle (ALM) using *M. franciscanus* tissue from the 2010 collection. Comparison of log2 fold-change in gene expression with age for the selected genes determined by qRT-PCR (open bars) and RNA-Seq (solid bars). The black bars represent RN and the red bars represent ALM. Gene names: Tnks1 – Tankyrase-1, Capn – calpain, Wnt1 – Wingless-type MMTV integration site-1, Mab21 – Mab-21-like 2, Lkb1 – Liver Kinase B1/Serine Threonine Kinase 11, Hyou1 – Hypoxia up-regulated 1, Neurx – Neurexin, Glur6 – Glutamate receptor ionotropic kainite-like 2, MAP3K9 – mitogen activated protein kinase kinase kinase 9, EBP – echinenone-binding protein, SM30E – spicule matrix protein 30E, MstnB – myostatin B.

Twelve genes were selected for verification by quantitative RT-PCR (9 for RN and 3 for ALM) using RNA from individual sea urchins collected in 2010. In all cases the qRT-PCR expression levels were significantly different between the Small (young) and Large (old) sea urchin groups and all genes showed the same trend of increased or decreased expression when compared to the RNA-Seq results, although the magnitude of the change was different in some cases (Fig. 2; Supplementary Table S1).

### Functional analysis of differentially expressed genes in Aristotle’s lantern muscle (ALM)

**Figure 3:**
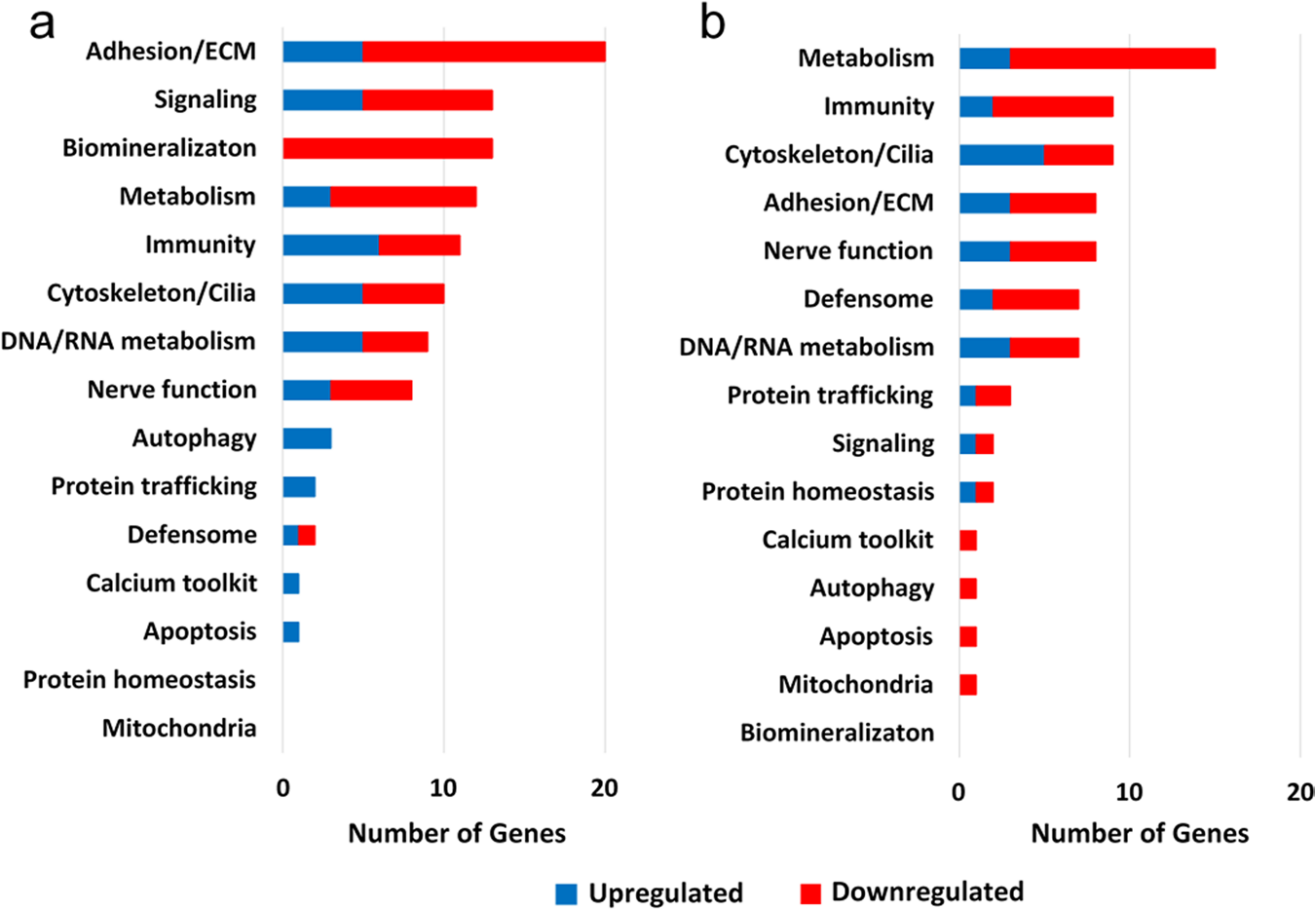
Distribution of genes upregulated and downregulated with age in *M. franciscanus* Aristotle’s Lantern Muscle (**a**) and Esophagus (**b**) curated into modified sea urchin custom gene ontology categories. Blue and red bars represent the number of genes in each category upregulated and downregulated, respectively.

The expression of 144 genes was significantly altered with age in ALM (65 upregulated and 79 downregulated) [log2 fold-change < −1 or >1, q-value (FDR) < 0.05]. Thirty-nine genes encoded uncharacterized proteins and the remaining 105 genes were assigned to functional categories based on a modified version of the custom sea urchin gene ontology of Tu *et al*.^27^ (Fig. 3a; Supplementary Table S2). The functional categories most affected with age were Adhesion/ECM (extra cellular matrix), Signaling, Biomineralization, Metabolism, Immunity, and Cytoskeleton/Cilia, each with 10 to 20 genes significantly altered. In the Signaling category, there was an upregulation with age in expression of the gene encoding myostatin (GDF8, Growth differentiation Factor 8) a member of the Transforming Growth Factor beta superfamily that inhibits muscle growth^28^. This is consistent with the slowed growth rate observed with increasing size of sea urchins^19^. There was a decrease in expression of 13 genes whose products play a role in biomineralization (Table S2). The presence of biomineralization gene transcripts may be the result of sclerocytes that co-isolated with muscle tissue when it was scraped from the pyramids of Aristotle’s lantern. It is interesting that no genes associated with mitochondrial function or protein homeostasis were significantly altered with age in ALM.

### Functional analysis of differentially expressed genes in esophagus (ES)

The expression of 123 genes was significantly altered with age in ES (53 upregulated and 70 downregulated) [log2 fold-change < −1 or >1 level, q-value (FDR) < 0.05]. Of the 123 differentially expressed genes, 49 encoded uncharacterized proteins and 74 were assigned to functional categories using the modified custom sea urchin gene ontology of Tu *et al*.^27^ (Fig. 3b; Supplementary Table S3). The category of “Metabolism” was most affected with 3 upregulated and 12 downregulated genes. Nine genes in the Cytoskeleton/Cilia category were altered with age including upregulated expression of 4 genes associated with cilia formation and function [dynein regulatory complex protein 9 (Iqcg), Cilia and Flagella Associated Protein 126 (CFAP126), Cilia and Flagella Associated Protein 69 (CFAP69), sperm-associated antigen 8 (Spag8)]. In sea urchin larvae, the proximal zone of the esophagus is densely ciliated to assist in the transport of food^29^, a function that may be preserved with age in adults. Only 1 gene was significantly altered with age in each of the Mitochondria, Apoptosis, Autophagy, and Calcium Toolkit categories, and only 2 genes were altered with age in the Protein Homeostasis and Signaling categories (Fig. 3b; Supplementary Table S3).

### Function and pathway analysis of differentially expressed genes in radial nerve cord (RN)

**Figure 4:**
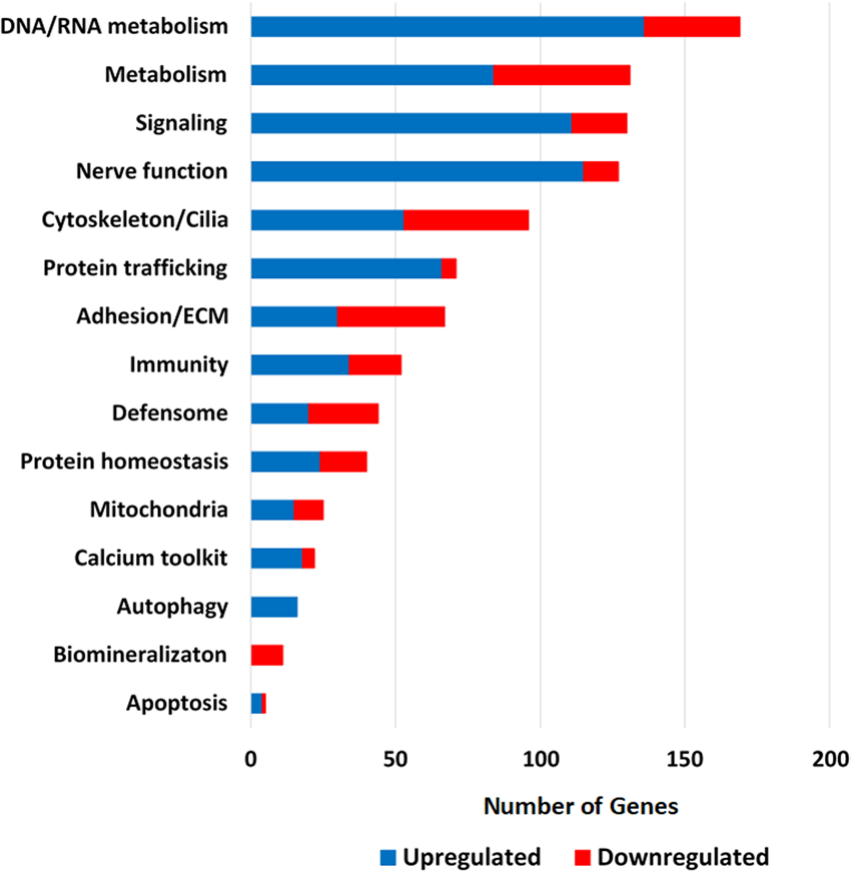
Distribution of genes upregulated and downregulated with age in *M. franciscanus* radial nerve cord (RN) curated into sea urchin custom gene ontology categories. Blue and red bars represent the number of genes in each category upregulated and downregulated, respectively.

The expression of 3,370 genes in RN was significantly altered with age at the log2 fold-change < −1 or >1 level [q-value (FDR) < 0.05] (2,291 upregulated and 1,079 downregulated) (Supplementary Table S4). The genes whose expression was most altered with age were selected for more detailed analysis including all genes that exhibited a log2 fold change of <−2 or >2. Using this criterion, the expression of 1,292 genes was significantly changed with age (907 upregulated and 385 downregulated). The differentially expressed RN gene sequences were assigned to functional categories based on a modified version of the custom sea urchin gene ontology of Tu *et al*.^27^ (Fig. 4; Supplementary Table S5). This resulted in 1,006 out of 1,292 differentially expressed RN genes being assigned to functional groups (726 upregulated and 280 downregulated), with the remaining 286 genes (181 upregulated and 105 downregulated) being uncharacterized. The most dramatic expression differences were in the categories of DNA/RNA Metabolism, Signaling, Metabolism, and Nerve function (Fig. 4).

The expression of 169 genes in the category “DNA/RNA metabolism” was significantly altered with age (136 upregulated and 33 downregulated) (Fig. 4; Supplementary Table S5). Forty-three of these genes encode known transcription factors, co-activators, or repressors (28 upregulated and 15 downregulated) and an additional 21 zinc finger proteins were upregulated with age. Several of the transcription factors are known to play a role in nerve function or neurogenesis [i.e. upregulated: Brain-2 (Brn1/2/4), Single-minded (Sim), Tailless (Tll), Forkhead Box P1 (FoxP), Prospero Homeobox 1 (Prox1), T-Box Transcription Factor 6 (Tbx6), and downregulated: Forkhead Box G1 (FoxG), Scratch Family Transcriptional Repressor (ScratchX), Paired Box 6 (Pax6), Homeobox 8 (Hox8), BarH-like homeobox (Barx)]. Twenty-nine genes associated with chromatin modification were significantly altered with age (25 upregulated and 4 downregulated). The upregulated genes encode 2 proteins that modulate DNA methylation, 9 proteins that modulate histone methylation, acetylation and ubiquitination and 10 proteins with a role in chromatin remodeling. Three homologues of histone protein encoding genes were upregulated (EhH1_40, EhH2a_67, EhH4_81) and 3 were downregulated (HH2az, H1-0, H3.3) with age. Eight genes known to play a role in DNA repair were altered with age (6 upregulated and 2 downregulated) and 2 genes involved in telomere maintenance were upregulated with age. There were 7 upregulated genes that play a role in transport across the nuclear envelope [i.e. Importin 5 (Kpnb3), Importin 4 (Ipo4), Nucleoporin 210 (Nup210), Exportin 6 (Xpo6), Exportin 7 (Xpo7), Translocated Promoter Region (Tpr), RAN Binding Protein 3 (Ranbp3)]. Twenty-six genes associated with RNA metabolism were significantly altered with age (18 upregulated and 8 downregulated). The expression of 17 genes involved in rRNA, tRNA and mRNA processing and turnover was upregulated while the expression of five genes that play a role in splicing was downregulated.

A large number of age-related differentially expressed genes were associated with the “Nerve Function” category (115 upregulated and 12 downregulated) (Fig. 4; Supplementary Table S5). Many genes that play a role in maintenance of nerve function and neuroprotection were altered with age (38 upregulated and 4 downregulated) including an upregulation of the nerve growth factor Neurotrophin (Nt) and Neurotrophic Tyrosine Receptor Kinase (Trk). Twelve genes associated with synaptogenesis and 18 genes associated with axonogenesis, neurogenesis, and neurite outgrowth were up-regulated with age (Table 2). Many genes associated with neurotransmitter production, transport and function were altered with age (45 upregulated and 8 downregulated). The neurotransmitter receptors affected by age are shown in Table 2 and include metabotropic glutamate receptors (5 upregulated) and ionotrophic glutamate receptors (3 kainate type upregulated, 3 AMPA type downregulated). Three GABA receptors and regulators of GABA production (Clptm1 and Gad) as well as one glycine receptor and a transporter that removes glycine from the synaptic cleft (Glyt) were also upregulated. Ten genes associated with acetylcholine (ACh) neurotransmission were altered with age (2 muscarinic ACh receptors and 7 nicotinic ACh receptors were upregulated while 1 nicotinic ACh receptor was downregulated) (Table 2), as well as genes involved in choline transport and metabolism [High Affinity Choline Transporter 1 (Slc5a7_1) and Acetylcholinesterase (AchE), both upregulated]. Biogenic amine neurotransmitters were also affected with age including an upregulation of an adrenergic receptor subunit and modulators of adrenergic receptor activity [beta-arrestin 1 (Arrb1) and alpha-arrestin 3 (Arrdc3)] as well as dopamine receptor and dopamine metabolism [Dopamine beta-hydroxylase (Dbh) and tyrosine hydroxylase (Th)]. Serotonin receptor 1a was upregulated while receptor 1b was downregulated with age, and a melatonin receptor was downregulated (Table 2). Several neuropeptide receptors were upregulated with age including neuropeptide S receptor, somatostatin receptors, galanin receptor, tachykinin receptor and octopamine receptor (Table 2). A number of the genes assigned to the “Calcium Toolkit” category are involved in the regulation of neuronal signaling pathways, including the downregulated genes: Calmodulin Like 3 (CalmL3), Calpain (Capn), Regucalcin (Rgn), and upregulated genes: Transient Receptor Proteins (Trpc5_4, Trpc5_6, Trpv6L, Trpc7) and Doublecortin Like Kinase 1 (Dclk1L). Additional upregulated genes in this category are involved in calcium homeostasis, neuronal excitability, and excitation-contraction coupling or light sensing (Supplementary Table S5).

**Table 2:** Nerve function genes differentially expressed in *M. franciscanus* radial nerve cord.

Nerve Function	Gene name	Gene ID	Log2 FC with age
**Synaptogenesis**			
Neurexin	Neurx	SPU_024416	3.13
Synaptotagmin 17	Syt17	SPU_001702	2.88
Synaptotagmin 12	Syt12	SPU_012856	2.15
Syntaxin 1 A	Stx1	SPU_012329	2.46
Syntaxin 6	Stx6	SPU_001979	2.20
Syntaxin binding protein 1	Stxbp1	SPU_002552	3.03
Piccolo	Pclo	SPU_012692	2.24
Adaptor-related protein complex 3, beta 2–2	Ap3b2–2	SPU_004682	2.31
Neuroligin-4	Nlgn4yL	SPU_016260	2.62
Leucine Rich Repeat Transmembrane Neuronal	Lrrtm2	SPU_025906	3.51
Synapse defective	Syde	SPU_001939	3.34
muscle skeletal receptor tyrosine kinase	MuskL	SPU_024610	2.38
**Axonogenesis/Neurogenesis**			
Semaphorin	Sema	SPU_010331	2.25
Semaphorin-1A	Sema1A	SPU_025927	3.68
Semaphorin 6D	Sema6d	SPU_001892	2.70
Plexin A2	PlexA2	SPU_009600	2.44
Prune homolog	Pruneh	SPU_007665	3.63
Neurofascin	L1	SPU_021428	3.47
Latrophilin 3	Lphn3	SPU_006950	3.43
glycerophosphodiester phosphodiesterase 5	Gdpd5	SPU_020578	3.38
PTPRF Interacting Protein Alpha 1 (Liprin)	Ppfia	SPU_026548	3.27
cadherin EGF LAG seven-pass G-type receptor 1	Celsr1	SPU_015321	2.88
Cdk5 and Abl enzyme substrate 1	Cables1	SPU_000952	2.47
Serine/Threonine-Protein Kinase ULK4	Ulk4	SPU_008621	2.39
Fasciculation and Elongation Protein Zeta 2	Fez2	SPU_018968	2.32
Down Syndrome Cell Adhesion Molecule	DscamL	SPU_009818	2.21
FYVE domain containing 27-like	FYVED_27	SPU_026865	2.19
roundabout homolog	RoboL	SPU_008663	2.13
Netrin1	netrin1	SPU_004245	2.08
Rap guanine nucleotide exchange factor 2	Rapgef2	SPU_004097	2.04
**Neurotransmitter Receptors**			
Glutamate receptor (ionotropic)	Glur2	SPU_ 013765	2.35
	Glur6	SPU_028455	2.44
	Glur6_1	SPU_ 014664	3.82
	Glur6_2	SPU_008910	3.57
	Glur1	SPU_ 027899	−2.77
	Gria1	SPU_ 008971	−2.74
	Glur3	SPU_ 002787	−2.47
Glutamate receptor (metabotropic)	Mglur3	SPU_ 025315	2.31
	Mglur3_2	SPU_ 008988	3.12
	Grm3	SPU_007747	2.63
	Grm6L	SPU_ 026773	2.67
	Grm8	SPU_ 018478	3.02
Gamma-aminobutyric acid (GABA) receptor	Gabbr1_3	SPU_ 004567	2.64
	Gabbr2L	SPU_ 028211	2.63
	Gpr51_1	SPU_ 018620	2.51
Glycine receptor	Glra1_1	SPU_ 017211	3.22
Acetylcholine receptor (muscarinic)	MachrM4	SPU_ 019228	3.68
	Chrm3L	SPU_ 021092	2.04
Acetylcholine receptor (nicotinic)	Chrna1L_1	SPU_ 011871	2.34
	Nacha2	SPU_ 002737	2.40
	Nacha7	SPU_ 011220	3.31
	Nacha7_1	SPU_ 013095	2.09
	Chrna7_8	SPU_ 026876	2.27
	Chrna9_2	SPU_ 000767	3.62
	Chrna10	SPU_ 027434	2.78
	Chrna9	SPU_ 017132	−2.23
Adrenergic receptor	Adrb1L_3	SPU_011123	2.22
Serotonin (5-hydroxytryptamine) receptor	Htr1a	SPU_ 014269	3.24
	Htr1b	SPU_005967	−3.29
Dopamine receptor	Drd1L_1	SPU_ 010497	2.28
Melatonin receptor	Mtnr1b	SPU_ 020142	−2.71
Neuropeptide S receptor	NpSLr	SPU_021291	3.29
Somatostatin receptor	Sstr1L	SPU_003125	2.32
	Sstr4L	SPU_005595	2.22
	Sstr5L	SPU_005473	2.21
	Sstr5L_2	SPU_024300	2.76
Galanin receptor	Galr2L_36	SPU_028056	2.80
Tachykinin receptor	Tacr2	SPU_023473	2.04
Octopamine receptor	Octr1	SPU_024934	2.62

The expression of 66 genes encoding products assigned to the “Protein Trafficking” category was upregulated while 5 were downregulated with age (Fig. 4; Supplementary Table S5). The upregulated genes include 7 that play a role in post-Golgi transport and secretory or synaptic vesicle function and 7 genes that play a role in ER and Golgi structural organization and stability. There was also an increase in expression of five members of the ER stress response that clears misfolded proteins from the ER [i.e. Membralin (Mbr1), UDP-Glucose Glycoprotein Glucosyltransferase 1 (Ugcgl1), Thioredoxin Domain Containing 5 (Txndc5), Tripartite Motif Containing 13 (Trim13), Eukaryotic Translation Initiation Factor 2 Alpha Kinase 3 (Eif2ak3perk)].

A large number of genes associated with the “Cytoskeleton/Cilia” category was modulated with age (53 upregulated and 43 downregulated) including genes that play a role in axon formation and neuronal migration (Fig. 4; Supplementary Table S5). Upregulated genes are involved in maintenance of subcellular spatial organization and integrity, cell motility, and organelle positioning including 26 genes associated with cytoskeletal structure and dynamics, and 16 genes encoding molecular motors for intracellular transport of vesicles and organelles (e.g. 10 kinesins, 2 dyneins, 4 myosins). Many genes encoding proteins associated with cilia formation and function were altered with age (5 upregulated and 15 downregulated). Although the role of cilia in the nervous system of adult sea urchins is not well characterized^30^, there is increasing evidence in humans that primary cilia may play a role in neural stem cell activity, neurogenesis, neuronal maturation, and maintenance^31^.

Numerous genes associated with the adhesion and extracellular matrix category “Adhesion/ECM” were modulated with age (30 upregulated and 37 downregulated) indicating active ECM remodeling (Fig. 4; Supplementary Table S5). Ten genes encoding structural components of the ECM were upregulated while 21 were downregulated including 7 collagen genes and lysyl oxidase (LoxL2) which catalyzes the cross-linking of collagen molecules. Eight genes that play a role in cell-cell or cell-matrix adhesion were downregulated with age, while 14 genes were upregulated including 3 integrins and 1 cadherin. Five genes encoding matrix metalloproteases were increased in expression with age while five genes encoding tissue inhibitors of metalloproteinase (TIMP) were downregulated.

A large number of genes encoding components of the signaling pathways category “Signaling” were altered with age in RN (111 upregulated and 19 downregulated), representing a wide range of cellular processes including cytoskeletal organization, vesicle exocytosis, cell adhesion, cell survival, and protein synthesis (Fig. 4; Supplementary Table S5). G-protein coupled receptors (GPCR) were among the most affected with 29 upregulated and 1 downregulated. Downstream of GPCR, genes associated with cAMP dependent signal transduction (4 upregulated and 1 downregulated) and upregulation of A-Kinase Anchoring Protein 1 (Akap1) which modulates protein kinase A activity were altered with age. In addition, four genes encoding proteins in the phosphoinositide phospholipase C (PLC) and protein kinase C (PKC) pathways were upregulated with age. Other pathways affected with age were the Ras GTPase family members (16 upregulated and 2 downregulated) and components of the mitogen-activated protein kinase (MAPK) pathway (9 upregulated).

Several genes associated with the Wnt signaling pathway (3 upregulated and 5 downregulated), the Notch signaling pathway (2 upregulated), and the TGFβ/BMP signaling pathway (4 upregulated and 2 downregulated) were affected with age (Supplementary Table S5). Only 3 genes that play a role in insulin signaling were altered with age in red sea urchin nerve tissue: an insulin-like growth factor binding protein (IgfalsL) was upregulated while an insulin-like growth factor binding protein (IgfbpL7) and sorbin and SH3 domain containing 1 (Sorbs1) were downregulated (Supplementary Table S5).

Gene expression patterns indicated age-related modulation of several genes associated with the 5’ AMP-activated protein kinase (AMPK) and mechanistic target of rapamycin (mTOR) pathways (Supplementary Table S5). This included upregulation in expression of Phosphatase and Tensin Homolog B (PtenB) and Inositol Polyphosphate-5-Phosphatase F (Inpp5f), which negatively regulate AKT (an upstream positive regulator of mTORC1), and Serine/Threonine Kinase 11 (Lkb1/STK11) which phosphorylates and activates AMPK as well as Prkag2, the regulatory subunit of AMPK. It should be noted that expression of Ulk1 (Unc-51 like autophagy activating kinase 1) which plays a role in facilitating AMPK induced autophagy and inactivation of mTORC1 was increased with age in the red sea urchin RN tissue at log2 fold-change of 1.65 (Supplementary Table S4, SPU_027245). AMPK inhibits mTORC1, but has been shown to activate mTORC2^32^. mTORC2 plays a role in cytoskeletal organization and cell migration and Mip1 (Sin1), a component of mTORC2, was upregulated in sea urchin RN with age.

In the “Protein Homeostasis” category, 14 ribosomal protein genes were downregulated, while there was an upregulation of translation initiation factor Eif4e3, a negative regulator of translation of target mRNAs^33^ and Eif2ak3perk which phosphorylates and inactivates EIF2 leading to reduced global protein synthesis (Supplementary Table S5). In addition, 15 genes involved in ubiquitin-proteasome function increased in expression suggesting enhanced protein degradation in RN with age (Supplementary Table S5).

Sixteen genes encoding lysosomal proteins were upregulated with age including many known to play a role in autophagy (Fig. 4; Supplementary Table S5). These include upregulation of Atg14 (Autophagy-related 14 or Becn1-associated) a key regulator of autophagy, Ubiquitin Specific Protease 36 (Usp36) that plays a role in activation of autophagy, phosphatidylinositol 3-phosphate-binding protein (Wdfy3) that functions as a master conductor for aggregate clearance by autophagy, as well as RB1-inducible coiled-coil 1 (Rb1cc), Tectonin Beta-Propeller Repeat Containing 1 (Tecpr1), Lysosomal Associated Membrane Protein 1 (Lamp1), and Estrogen-Induced Gene 121-Like Protein (Kiaa1324L) which regulate events of autophagosome formation and function, and autophagin-1 (Atg4hb) which encodes a cysteine protease required for autophagy.

The expression of 25 genes classified as “Mitochondria” were significantly altered with age (15 upregulated and 10 downregulated) (Fig. 4; Supplementary Table S5). Three genes associated with oxidative phosphorylation were downregulated [NADH:Ubiquinone Oxidoreductase Subunit B1 and subunit A11 (NdufB1 and NdufA11), ATPase Subunit D (Atp5h)] and there was an upregulation of Pyruvate Dehydrogenase Kinase (Pdk2) which phosphorylates and inactivates Pyruvate Dehydrogenase, reducing aerobic respiration and thereby playing a key role in the regulation of glucose homeostasis. Three of the upregulated mitochondria genes [Mitochondrial Elongation Factor 1 (Smcr7L), Phosphatidic acid-preferring phospholipase A1 (Ddhd1) and Mitochondrial Dynamin Like GTPase (Opa1)] are associated with mitochondrial morphology, cytoplasmic distribution and fission, and also upregulated was the catalytic subunit of mitochondrial DNA polymerase (Polg).

Expression of 44 genes associated with the chemical “Defensome” was altered with age, including 20 upregulated and 24 downregulated (Fig. 4; Supplementary Table S5). Among the upregulated genes were 10 members of the ATP-binding cassette transporters (2 AbcA, 3 AbcB, 4 AbcC, 1 AbcG family members) that transport various molecules across extra- and intra-cellular membranes and play an important role in efflux of xenobiotic compounds. Seven chaperones or co-chaperones were upregulated with age, including hypoxia upregulated 1 (Hyou1), which belongs to the heat shock protein 70 family and has a role in cytoprotective cellular mechanisms triggered by oxygen deprivation. Among the downregulated genes were several that play a role in xenobiotic metabolism, conjugation, and elimination including 6 members of the cytochrome P450 family, 2 UDP-glucuronosyltransferases, 2 sulfotransferases, 6 glutathione S-transferases, and an organic cationic transporter.

In the “Metabolism” category there were 84 upregulated and 47 downregulated genes including genes involved in amino acid, carbohydrate, and lipid metabolism, as well as a variety of other specialized metabolic functions (Fig. 4; Supplementary Table S5). Genes involved in lipid metabolism including fatty acid, cholesterol, and sphingolipid metabolism were among the most affected with 28 upregulated and 9 downregulated. Six genes encoding amino acid transporters were upregulated with age, while 5 genes encoding monocarboxylic acid transporters were downregulated. Hypoxia Inducible Factor Prolyl 4-Hydroxlase (PH-4) was among the downregulated genes and Iodothyronine deiodinase type I (Dio1) showed the largest reduction in expression with age (−5.2 log2 fold-change). Iodothyronine deiodinase is important for regulation of thyroid hormones essential for growth, development and basal metabolism and is known to play a role in development and metamorphosis in sea urchins^34^. Reduced expression of iodothyronine deiodinase is consistent with the slowed growth and decreased metabolic rate that occurs in sea urchins with increase in size^19,35–37^.

The expression of 52 genes associated with “Immunity” was altered with age (34 upregulated and 18 downregulated) (Fig. 4; Supplementary Table S5). Among the upregulated genes there were 11 Scavenger Receptor Cysteine-Rich (SRCR), 7 NACHT and LRR containing proteins (NLR), 3 Toll-like receptors (TLR), 2 lectins, 2 macrophage MHC receptor 2-like genes, and 7 additional genes involved in innate immune cell signaling and antiviral response. Among the downregulated genes there were 6 carbohydrate binding lectins that play a role in recognition of pathogens, apoptotic and necrotic cells. Also downregulated were 5 genes encoding SRCR proteins, 1 TLR, 2 antimicrobial peptides, and amassin, a protein that facilitates clotting of coelomocytes.

Only 5 genes whose products play a role in “Apoptosis” were significantly altered with age (Fig. 4; Supplementary Table S5). There was an upregulation of two isoforms of Apoptotic Protease-Activating Factor (Apaf), caspase-3 substrate Gas2 that alters the actin cytoskeleton during apoptosis, and an inhibitor of apoptosis (Kiaa0317L). Downregulated was the pro-apoptotic protein Interferon Alpha Inducible Protein 27 (Ifi27) that promotes INF-induced apoptosis.

Eleven genes in the “Biomineralization” category were downregulated with age in RN tissue (Fig. 4; Supplementary Table S5). Whether these genes are expressed in nerve tissue or due to sclerocytes that co-isolated with the RN tissue was not investigated.

### Ingenuity Pathway Analysis (IPA) of differentially expressed *M. franciscanus* RN genes

**Figure 5:**
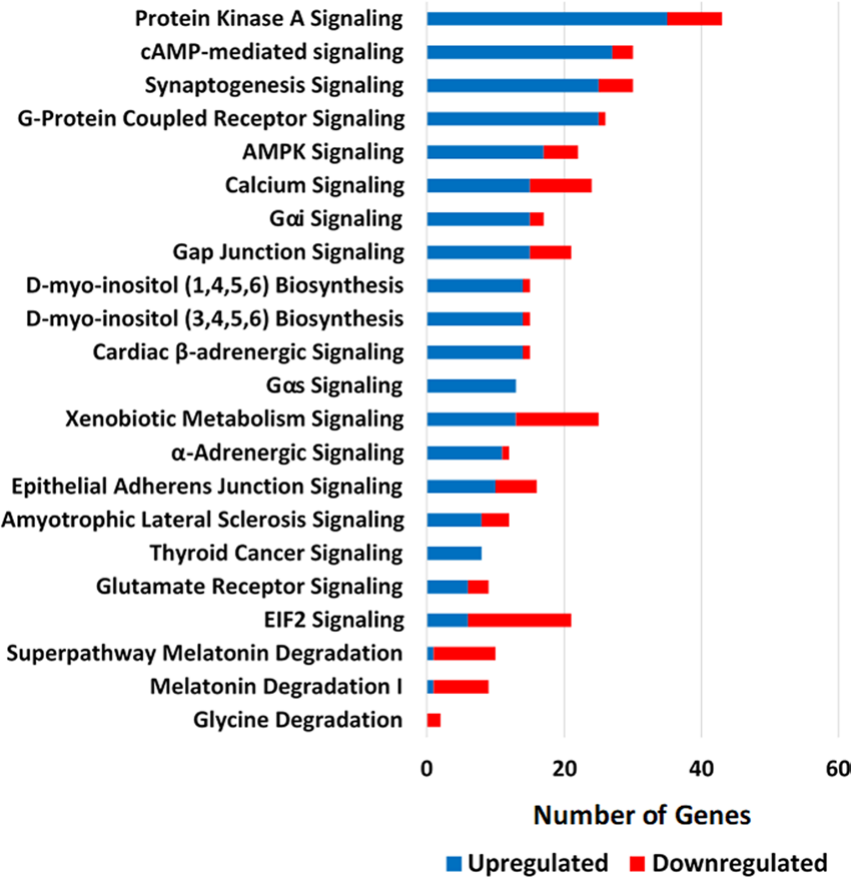
IPA canonical pathways significantly affected by age in sea urchin radial nerve cord (BH FDR adj P-value <0.05). Blue and red bars represent the number of genes in each pathway upregulated and downregulated, respectively.

IPA was used as an additional approach to identify canonical pathways, networks, and functions associated with the age-related differentially expressed genes in sea urchin RN tissue [log2 fold-change < −2 or >2 level, q-value (FDR) < 0.05]. Human gene identifiers corresponding to the differentially expressed sea urchin RN genes were used as input for IPA pathway analysis (Supplementary Table S6). A total of 1,038 out of 1,042 input gene identifiers mapped in IPA and identified 22 significantly enriched pathways with age (BH FDR adj P-value < 0.05) (Fig. 5; Supplementary Table S7). The pathways most affected included signaling pathways associated with nervous system function such as PKA, cAMP, GPCR, calcium, and synaptogenesis signaling. In addition, AMPK signaling, D-myo-inositol-tetrakisphosphate signaling, xenobiotic metabolism signaling, and EIF2 signaling were amongst the top 22 affected pathways.

Of the 22 pathways significantly affected by age, 10 had a z-score ≥ 2.0 or ≤ −2.0 suggesting overall activation or inhibition of the pathway, respectively (Supplementary Table S7). These included the following activated pathways: cAMP, synaptogenesis, Gαs, α-adrenergic signaling, and D-myo-inositol-tetrakisphosphate biosynthesis. The inhibited pathways include: EIF2 signaling and melatonin degradation pathways. Overall, the IPA results were consistent with those from curation of the differentially expressed genes into the custom sea urchin ontology groups, providing an additional validation of the results.

### Comparison of age-related gene expression between sea urchin tissues and species

At the log2 fold-change level of < −1 and >1, 68 of the 144 differentially expressed genes in ALM, and 42 of the 123 genes in ES were also differentially expressed in RN (Supplementary Table S2; Supplementary Table S3). Only 3 genes were shared exclusively between ES and ALM and only 3 genes were shared between all 3 tissue types (Supplementary Table S3). Two of the common genes encode uncharacterized proteins (SPU_001941 and SPU_014904) and the third gene encodes Solute Carrier Family 16, Member 12-like-2 (Slc16a12L_2), a monocarboxylic acid transporter that mediates creatine transport across the plasma membrane, which showed decreased expression with age in all three tissue types.

The age-related pattern of gene expression in RN of the red sea urchin was compared to that previously reported for the purple sea urchin *S. purpuratus*^23^. Microarray analysis revealed 177 age-related differentially expressed genes in *S. purpuratus* RN^23^ and comparison with RN of *M. franciscanus* revealed 27 genes in common (Supplementary Table S8). Four of these genes encode proteins with unknown function and 6 of the genes showed the opposite expression in *M. franciscanus* versus *S. purpuratus* RN. The common downregulated genes include Homeobox transcription factor (Hlx), HIF-Prolyl 4-Hydroxylase (Ph4), Parkin Coregulated Chaperone-Binding Protein (Pacrg), Collagen-Fibrillar V/XI (6Afcol), and Wnt1. The common upregulated genes were the transcriptional repressor Scratch, Prokineticin Receptor-2 (Prokr2), KIAA0391-protein-like that functions in mitochondrial tRNA maturation, and Syntaxin 1 A (Stx1) that plays a role in the docking of synaptic vesicle with the presynaptic plasma membrane. The limited number of overlapping genes that behave similarly with age in RN of these two sea urchin species may, at least in part, be the result of technical differences in the two approaches used to evaluate gene expression (microarray versus RNA-Seq) and the elimination of low expressors from the microarray analysis.

## Discussion

It has been proposed that lifelong transcriptional stability is a key determinant of longevity and negligible senescence^24^. Consistent with this idea, muscle and esophagus tissue from the long-lived red sea urchin *M. franciscanus* showed a high level of transcriptional stability with a limited number of significant age-related changes. In contrast, radial nerve cord (RN) showed more than 3,000 age-related differentially expressed genes at a two-fold level, and 1,292 at a four-fold level, representing a broad range of cellular functions and pathways including nerve function, signaling, metabolism, and DNA maintenance. Altered expression of many genes involved in transcriptional regulation, epigenetic modification, chromatin structure and remodeling indicated a strong transcriptional response to aging in sea urchin RN tissue. Transcriptional studies on six species (entire organisms for *C. elegans* and Drosophila and brains of mice, rats, chimpanzees and humans) found an age-associated decrease in expression of genes involved in synaptic transmission, vesicular transport, calcium homeostasis, mitochondrial function and an induction of expression of genes associated with stress-response such as DNA repair, antioxidant defense and immune function^38,39^. The observation that many of the transcriptional changes observed in the red sea urchin nerve tissue differ from those observed in mammals and invertebrate model organisms suggests a novel age-related transcriptional program in these long-lived animals; however, whether the changes represent an orchestrated transcriptional program or are in response to stochastic events remains to be determined.

A loss of synaptic integrity and decline in synaptic gene expression is characteristic of the aging nervous system across phyla^39–41^. In the aging human frontal cortex, genes involved in synaptic plasticity that underlie learning and memory are among the most significantly affected^38^ including the reduced expression of genes encoding subunits of ionotropic and metabotropic glutamate receptors as well as the GABA receptor^38^. Similar to other animals, the sea urchin RN showed an age-related decrease in expression of three AMPA glutamate receptor subunits. In contrast, there was an upregulation in the expression of several subunits of metabotropic glutamate receptors, ionotropic (kainate) glutamate receptors, and one AMPA glutamate receptor. In addition, there was an increase in expression of receptor subunits and regulators of GABA, glycine and acetylcholine neurotransmission as well as biogenic amine and neuropeptide neurotransmitters. Therefore, aging sea urchin neurons appear to be upregulating expression of numerous genes involved in neurotransmission, while downregulating specific subunits of AMPA, serotonin, and melatonin receptors.

In addition to neurotransmitters, there was increased age-related expression of numerous genes with a role in synaptogenesis, axonogenesis, and neuroprotection, including an upregulation of the nerve growth factor Neurotrophin and Neurotrophic Tyrosine Receptor Kinase associated with neuronal maintenance and survival. There was also modulation in expression of many genes involved in cytoskeletal dynamics and vesicular transport in aging sea urchin RN tissue. Many of the upregulated genes were involved in Golgi function, post-Golgi transport, secretory vesicle function, and synaptic vesicle cycling and localization. The cytoskeleton genes upregulated with age in sea urchin RN tissue are involved in maintenance of subcellular spatial organization and integrity, cell motility, and organelle positioning (e.g. ankyrin, spectrin) and also genes encoding molecular motors for intracellular transport of vesicles and organelles (e.g. kinesin, dynein). The neuronal cytoskeleton is required for axon formation and axonal transport but also provides the structural basis for several specialized axonal structures at the synapses essential for localizing the synaptic vesicles to the plasma membrane^42^. A number of neurodegenerative diseases have been linked to defective axonal cytoskeletal regulation^42^ and the upregulation of key cytoskeletal genes in sea urchin nerve tissue suggests preservation of this aspect of nerve function with age.

Synaptic function and plasticity are dependent upon the regulation of neuronal calcium fluxes and calcium-mediated signaling pathways^39^. Calcium homeostasis and the synaptic calcium signaling system in the mammalian brain is affected by age with reduced expression of calmodulin 1 and 3, CAM kinase II and IV, calcineurin B, multiple protein kinase C isoforms, Na^+^/Ca^2+^ exchangers, voltage gated calcium channels (VGCC), transient receptor potential TRP receptors, and plasma membrane calcium ATPase^39,43^. In the red sea urchin RN tissue, many genes associated with calcium signaling showed the opposite trend with upregulation of a plasma membrane ATPase, two VGCC, three Na/Ca exchangers, four TRP receptors, and two protein kinase C isoforms, suggesting that the red sea urchin is able to effectively modulate calcium homeostasis and calcium signaling pathways to avoid the age-related changes that are typically seen in humans and other animals.

**Figure 6:**
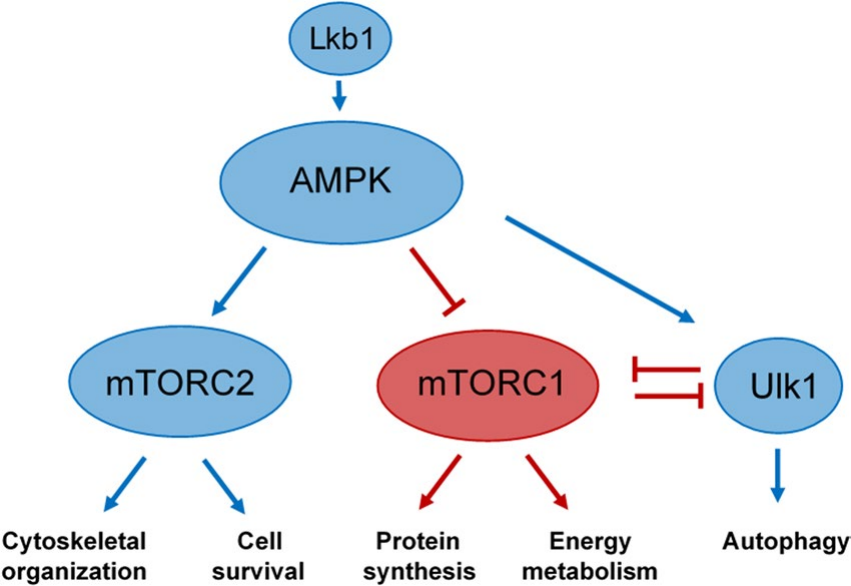
Schematic representation of the predicted activation of Lkb1, AMPK, Ulk1, and mTORC2 and inhibition of mTORC1 in sea urchin radial nerve tissue inferred from the age-related differential gene expression. Predicted increased activity is depicted in blue while predicted inhibition of activity is depicted in red with the predicted outcomes being increased autophagy, cytoskeletal rearrangement, and cell survival and decreased protein synthesis and mitochondrial energy metabolism.

Gene expression patterns indicated modulation of several genes associated with the AMPK and mTOR pathways which play an important role as master regulators of cellular metabolic homeostasis. These pathways respond to signals such as amino acid levels, bioenergetic status, hormone and growth factor signaling as well as oxidative stress, hypoxia, or DNA damage^44^. AMPK is a serine-tyrosine kinase that acts as the master metabolic regulator responding to cellular energy status (AMP/ADP:ATP ratio) to inhibit anabolic processes and stimulate catabolism^45^. mTOR forms two complexes, mTORC1 and mTORC2, which differ in their composition and downstream targets^46^. mTORC1 activity promotes protein, lipid, and nucleotide synthesis as well as mitochondrial energy production while inhibiting autophagy to support cell growth and proliferation^46^. mTORC2 plays a role in cytoskeletal organization and cell survival^32^. AMPK activation leads to inhibition of mTORC1 through direct and indirect mechanisms, and can lead to stimulation of mTORC2^32^. The predicted upregulation of AMPK and downregulation of mTORC1 in sea urchin RN tissue was supported by the observed downregulation in expression of a number of genes involved in protein synthesis as well as an upregulation in expression of genes involved in autophagy. Activation of AMPK or inhibition of mTORC1 by genetic, nutrient, or pharmacological manipulations increases longevity and confers protection against age-related pathologies to model organisms^45,47–49^. The improved healthspan and increased lifespan are thought to be largely due to increased levels of autophagy which serves to recycle cellular components to meet the biosynthetic and energy demands of the cell. Autophagy is known to play a protective role against onset of neurodegeneration in animal models and its downregulation in normal aging is thought to contribute to development of chronic neurodegenerative diseases^40,44,50^. Modulation of AMPK and mTOR pathways resulting in decreased cell growth and proliferation, and increased autophagy as illustrated in the model shown in Fig. 6, may serve to mitigate the detrimental effects of aging in the sea urchin nervous system.

Protein folding in the ER is a critical step in the biogenesis of most secretory and transmembrane proteins and the ER stress response or unfolded protein response (UPR) plays a fundamental role in the maintenance of cellular protein homeostasis^51^. UPR is characterized by the induction of chaperones to assist in the refolding of proteins, degradation of misfolded proteins by the proteasome, and attenuation of protein translation which is mediated by the serine-threonine kinase PERK^51^. PERK phosphorylates the initiation factor eIF2α thereby reducing global translation while at the same time activating translation of selected messages. Aging is typically associated with a decline in expression and activity of key UPR components leading to the accumulation of misfolded proteins and protein aggregates^51^. It is therefore interesting that sea urchin RN tissue showed an upregulation in expression of several genes whose products play a role in the ER stress response (i.e. Eif2ak3perk, Mbr1, Ugcgl1, Txndc5, Trim13). This was accompanied by an increase in expression of genes whose products play a role in the ubiquitin-proteasome pathway and autophagy and a decrease in protein synthesis. There was also increased expression of many chaperones in the “Defensome” category. The upregulation of UPR is expected to act as a protective process alleviating ER stress, however; prolonged ER stress has been shown to result in inflammatory signaling while unmitigated and excessive stress leads to apoptosis^51^. In red sea urchin RN tissue, the immune gene profile does not support a pro-inflammatory response and very few genes associated with apoptosis were altered with age.

The predicted increase in AMPK signaling and concomitant decrease in mTORC1 signaling and decreased global protein synthesis in aging sea urchin nerve tissue raises an important question. Synaptic function and plasticity rely on the expression of new proteins, and mTOR function has been reported to be critical to synaptic plasticity^52^. The solution to this problem may lie in the fact that there is localized protein translation in neurons. Localized protein synthesis is now well documented at synapses in both vertebrates and invertebrates (i.e. Aplysia) leading to the intriguing possibility that while overall protein synthesis is reduced via suppression of mTOR (resulting in the general benefits that promote longevity), local protein synthesis at the synapses may confer plasticity and preservation of neuronal function. The localized translation at synapses is accomplished by transporting mRNAs out of the nucleus where they bind to RNA-binding proteins. These RNP complexes are then translocated along microtubules via motor proteins to synapses where the mRNA is made available to the local translation machinery^53^. Further, it has been shown that Netrin1 plays a key role in promoting translation at sites of synaptic contact or synaptic stimulation^54^. Interestingly, one of the first players identified in this local protein translation was Microtubule-Associated Protein 2 (MAP2), the corresponding transcript for which was upregulated with age in sea urchin RN tissue. In addition, there was increased expression of molecular motors (kinesin, dynein), genes that encode proteins that play a role in export of mRNA from the nucleus (Tpr, Ranbp3, Nup210), and Netrin-1 in sea urchin RN tissue. This mechanism may provide a means to locally establish, maintain, and modify the synaptic proteome with age in the red sea urchin nerve tissue.

It has been proposed that the nervous system may act as a central regulator of aging by coordinating the physiology of extraneural tissues^40,55^. In support of this, manipulating longevity genes in the central nervous system of model organisms such as worms, flies, and mice is sufficient to counteract the effects of aging at a systemic level^55^. Promoting autophagy in the nervous system of adult Drosophila not only prevents the age-dependent accumulation of damage in neurons but also leads to increased lifespan^56,57^. It is therefore tempting to speculate that beneficial changes in the nervous system of the red sea urchin may have more widespread benefits for other issues and the organism as a whole.

There are several shortcomings of this study that should be considered in evaluating the outcome. This study only compares two time points during the life span of the sea urchins and therefore may be missing potentially important trends that occur over the life of these animals. In addition, the maximum life span potential of this animal is not known and therefore we cannot definitely say that the oldest animals are approaching their maximum limit. For each tissue, RNA was pooled from the young or old sea urchins for each of the respective collections prior to RNA-Seq analysis and interindividual variation was only assessed in a small number of genes used for qRT-PCR. It has been reported that pooling of samples can be beneficial when differential expression is the sole objective of the study and/or when biological variation is high relative to measurement error^58^, as is often the case with wild caught animals. Using the whole radial nerve cord, does not provide information about different cell types in the nervous system; a shortcoming that could be addressed with single-cell transcriptomics. Finally, one must consider that gene expression does not always correlate with protein production and function and so inferences about function based on gene expression should be viewed with caution.

The age-related pattern of gene expression in the nervous system of the red sea urchin tissues was unique compared to humans and short-lived model animals; however, there was some overlap in gene expression between different tissues and species of long-lived sea urchins. The underlying factors that drive the observed changes in gene expression in the red sea urchin remain to be identified, and it is unclear whether the changes represent a predetermined sequence of programmed transcriptional regulation or a transcriptional response to stochastic changes that occur over time. The unique age-related gene expression profile, including altered expression of a number of transcriptional regulators and chromatin modifiers, hints to a negligible senescence promoting program. However, this assertion requires verification, and functional verification is required to determine if the observed changes in gene expression act to preserve tissue function and mitigate aging in these animals.

## Methods

### Collection of *M. franciscanus* and RNA isolation from muscle, esophagus and nerve tissue

*M. franciscanus* individuals were collected from the east side of Kendrick Island, British Columbia, Canada (lat/long 49 07.571 N 123 41.548 W, depth range of 20–45 feet) between July 14^th^ and August 1^st^ during the summers of 2010, 2011 and 2013. Each year, 6 small animals (<5 cm test diameter) and 4–6 large animals (>15 cm test diameter) were collected. Following collection, sea urchins were left to acclimate in an outdoor, flow-through aquarium at the Pacific Biological Station in Nanaimo, BC for about one week to ensure all animals were exposed to a uniform environment prior to dissection. Muscle tissue from Aristotle’s lantern (ALM), esophagus (ES) and radial nerve cord (RN) were dissected from the sea urchins and immediately immersed in RNA later solution (Qiagen, Valencia, CA) and frozen at −80 °C.

RNA was extracted from the tissues using Trizol reagent (Invitrogen/Life Technologies, Carlsbad, CA) followed by the RNA clean-up protocol of the RNeasy mini kit (Qiagen) with a 15 minute DNase I digestion step. Total RNA concentrations were determined by measuring absorbance at 260 nm using an Agilent 8453 Spectrophotometer (Agilent, Santa Clara, CA) and RNA integrity was confirmed by electrophoresis on 1% formaldehyde agarose gels.

### RNA Sequencing, de novo *M. franciscanus* transcriptome assembly and differential gene expression

RNA from each tissue type and size category (ALM-S, ALM-L, ES-S, ES-L, RN-S and RN-L) from animals collected in a given year were pooled using an equal amount from each individual sea urchin to yield a total of 2 µg for RNA-Seq analysis. Pooled samples from each of the three sampling years (2010, 2011 and 2013) were treated as independent biological replicates resulting in a total 18 samples that were sent to Otogenetics Corporation (Atlanta, GA) for RNA-Seq analysis. Purity and integrity of the RNA were assessed using an Agilent Bioanalyzer and OD260/280, and cDNA preparation was conducted using the SMARTer cDNA synthesis kit (Clontech, Mountainview, CA). The cDNA was profiled using Agilent Bioanalyzer, and subjected to Illumina library preparation using NEBNext reagents (New England Biolabs, Ipswich, MA). The quality, quantity and size distribution of the Illumina libraries were determined using an Agilent Bioanalyzer 2100. Sequencing was conducted on an Illumina HiSeq-2000 sequencer (Illumina Inc. San Diego, CA) resulting in a minimum of 20 million paired-end 100 base pair (bp) reads per sample.

RNA-seq reads were quality filtered and trimmed using Trimmomatic version 0.38. Adapter and primer sequences were removed, and quality-based trimming was performed using a 4-base wide sliding window approach with an average quality threshold of 15. Only sequences greater than 50 base pairs after trimming were kept for de novo assembly.

De novo transcriptome assembly was done using Trinity version 2.8.5 using default settings. Benchmarking Universal Single-Copy Orthologs (BUSCO) analysis was performed using BUSCO v3 with the Metazoa (odb9) gene set to assess transcriptome completeness. The transcripts were annotated using reciprocal blast searches against the *S. purpuratus* (SPU) protein set with an e-value cutoff of 10^–4^. SPU protein sequences were downloaded from EchinoBase.org.

Sequencing reads were then aligned to the transcripts with Bowtie2 version 2.3.4.3 and quantified using RSEM version 1.3.0 through Trinity’s built-in quantification pipeline with the “align_and_estimate_abundance.pl” script. The Trinity utility script “abundance_estimates_to_matrix.pl” was used to generate count matrices for the samples for downstream expression analysis. Transcripts that were classified as the same SPU gene were grouped for differential gene expression (DGE) analysis. DGE analysis was performed with DESeq 2 version 1.24.0 to compare expression between young (small) and old (large) individuals. Differences between young (small) and old (large) samples were analyzed within each tissue type and year, as well as averaged over all three years, and results with q-value (FDR) < 0.05 were considered significantly different.

### Function and canonical pathway analysis

The age-related differentially expressed genes were manually curated into functional groups using the custom-built sea urchin gene ontology major functional classes^27^ with the following modifications: DNA replication, histone, transcription factor, cell cycle and ZNF were combined into one category called “DNA/RNA metabolism”, the categories of adhesion and metalloprotease were combined into one category called “Adhesion/ECM”, and GPCR Rhodopsin, GTPase, kinase, phosphatase, and signaling were combined into one category called “Signaling”. Three new categories relevant to aging biology “Mitochondria”, “Autophagy”, “Protein homeostasis” were added to this classification for a total of 15 major functional classes. Any gene predictions that were not assigned an identity in EchinoBase were subjected to BLAST searches of the NCBI Reference Sequences (RefSeq) collection in an attempt to assign an identity for further functional characterization, and those for which there was no significant match or have no characterized function, were classified as “uncharacterized”.

Ingenuity Pathway analysis (IPA; QIAGEN Inc., Redwood City. CA) was used to identify significant networks, functions, and canonical pathways altered with age in sea urchin radial nerve tissue^59^. For input into IPA analysis, the human ENSEMBL identifiers with highest homology to the sea urchin protein sequences were selected using BLASTP (Supplementary Table S6). P-values provided by IPA were adjusted to account for multiple testing using the Benjamini-Hochberg FDR method.

### Verification of differentially expressed genes using quantitative RT-PCR

Differential expression of selected genes was verified using quantitative reverse-transcription polymerase chain reaction (qRT-PCR) on the ABI 7300 Real Time-PCR machine using the SYBR Green detection system (Applied Biosystems, Foster City, CA, USA). RNA from individual sea urchin tissues was reverse transcribed using the High Capacity cDNA Reverse Transcription Kit (Applied Biosystems). Primers for qRT-PCR were designed using Primer Express software (version 3.0) (Applied Biosystems) based on sequences for *M. franciscanus* selected using the annotated *S. purpuratus* genes blasted against *M. franciscanus* short sequence reads in NCBI (accession SRX000089) and synthesized by Bio-Synthesis (TX, USA) (Supplementary Table S9). The expression level of genes was determined using the delta-delta-CT method and normalized using a normalization factor derived from the geometric mean of expression of three stably expressed control genes^60^. Up to 10 control genes (mAct, GAPDH, cyclophilin7, profilin, HPRT, ATub5, PARP1, RPL8, PCNA, B-tubulin2) were tested and the gene stability measure (M) was determined to select the three most stable control genes for radial nerve cord (ATub5, HPRT, PARP1) and muscle (PCNA, RPL8 and B-tubulin2), with M values <0.4^60,61^. No-template-controls were included on each reaction plate to check for contamination and negative controls consisting of untranscribed RNA (No-RT controls) were performed for each RNA extraction to check for genomic DNA contamination. All verifications were performed using RNA from the tissues of small (young) and large (old) individual sea urchins from the 2010 collection, the same RNA that was used for RNA-Seq analysis.

## Supplementary information

Supplementary inforamtion.

Supplementary inforamtion2.

Supplementary inforamtion3.

Supplementary inforamtion4.

Supplementary inforamtion5.

Supplementary inforamtion6.

Supplementary inforamtion7.

Supplementary inforamtion8.

Supplementary inforamtion9.

Supplementary inforamtion10.

## Data Availability

The RNA-Seq data have been deposited in NCBI’s Gene Expression Omnibus (GEO) and Sequence Read Archive (SRA). All data are associated with BioProject accession PRJNA562282. The raw sequence FastQ files can be found in SRA project SRP219667 (BioSamples: SAMN12637227-SAMN12637246). DGE count tables and transcript assembly are accessible through GEO Series accession number GSE136352 (https://www.ncbi.nlm.nih.gov/geo/query/acc.cgi?acc=GSE136352). All additional data is available in the Supplementary information.
