# Supplementary material for: Unique age-related transcriptional signature in the nervous system of the long-lived red sea urchin Mesocentrotus franciscanus
Source: Sci Rep. 2020 Jun 8;10:9182. doi: 10.1038/s41598-020-66052-3 (PMC7280269; doi:10.1038/s41598-020-66052-3)
Supplement: Supplementary file 1 — Supplementary inforamtion. [file 41598_2020_66052_MOESM1_ESM.docx]

**Unique age-related transcriptional signature in the nervous system of the long-lived red sea urchin *Mesocentrotus franciscanus***

Jennifer M. Polinski^1^, Nicholas Kron^2^ Douglas R. Smith^1^ and Andrea G. Bodnar^1*^

Author Affiliations

^1^Gloucester Marine Genomics Institute, 417 Main Street, Gloucester, MA 01930

^2^Department of Marine Biology and Ecology, Rosenstiel School of Marine and Atmospheric Science, University of Miami, 4600 Rickenbacker Causeway, Miami, FL 33149

*Corresponding author: [andrea.bodnar@gmgi.org](mailto:andrea.bodnar@gmgi.org)

**Supplementary Figure S1**

**Supplementary Figure S1**. Length distribution and assembly statistics for the assembled master transcriptome of *M. franciscanus.*

1. **Length distribution of the assembled master transcriptome for *M. franciscanus***
2. **Summary of the assembled master transcriptome for *M. franciscanus***

| **Parameter** | **Total** |
| --- | --- |
| Number of transcripts | 233,762 |
| Total transcripts length (bp) | 268,562,424 |
| Minimum transcript length (bp) | 278 |
| Maximum transcript length (bp) | 15,725 |
| Average transcript length (bp) | 1,148 |
| N50 (bp) | 1,919 |
| N90 (bp) | 443 |
| %GC | 38.58 |
