# Supplementary material for: Unique age-related transcriptional signature in the nervous system of the long-lived red sea urchin Mesocentrotus franciscanus
Source: Sci Rep. 2020 Jun 8;10:9182. doi: 10.1038/s41598-020-66052-3 (PMC7280269; doi:10.1038/s41598-020-66052-3)
Supplement: Supplementary file 10 — Supplementary inforamtion10. [file 41598_2020_66052_MOESM10_ESM.doc]

**Unique age-related transcriptional signature in the nervous system of the long-lived red sea urchin *Mesocentrotus franciscanus***

Jennifer M. Polinski^1^, Nicholas Kron^2^ Douglas R. Smith^1^ and Andrea G. Bodnar^1*^

Author Affiliations

^1^Gloucester Marine Genomics Institute, 417 Main Street, Gloucester, MA 01930

^2^Department of Marine Biology and Ecology, Rosenstiel School of Marine and Atmospheric Science, University of Miami, 4600 Rickenbacker Causeway, Miami, FL 33149

*Corresponding author: [andrea.bodnar@gmgi.org](mailto:andrea.bodnar@gmgi.org)

**Supplementary Table S9**

**Supplementary Table S9**. Quantitative RT-PCR primer sequences

Target Gene GENE ID Primer Sequences

Mab21 SPU_001059 (F) CATGTCACTTTGGGTCGAGTTTAT (R) CGTTTGAAACCGTGATCGAA

Tnks1 SPU_011795 (F) CTTTACACATCGCCGCAAGA (R) GCACCGAGCGTAATCAATTCA

Wnt1 SPU_011756 (F) GGCGGCTGCAGTGACAAC (R) TCGCCCGAGTCTACAAACTTC

capn SPU_023461 (F) CCACCAGGCCACTATGTCATT (R) CGCAACAGAAAGTCACCCTCTT

Lkb1/Stk11 SPU_022284 (F) GAATACCTGCACAGCCAGCAT (R) CCGGCGGTGGTAAGAAGAA

Hyou1 SPU_024402 (F) AAGCTGCTTGAGGTGAATCTGAA (R) TGAGCTTTGATTCCTTTGTTATCG

MAP3K9 SPU_018191 (F) ATGCGGCCCTGCTGAAG (R) AGGTTGAGTTCCCTCTCCAAGAG

Glur6 SPU_008910 (F) TGGCGAATCAGAACAAGATCTC (R) TGGTTTTTCCTCTTTTCGAAGAA

Neurx SPU_024416 (F) AATCCCTTCACCTTCAGAACAAAA (R) AGCGTCAGGGCTGGATAAGA

MstnB SPU_002795 (F) TCGAGGGTCGTGCGATTC (R) CCTCATAGTGTGCGCGTAGAAG

EBP SPU_013736 (F) TTCGACATCACGAGCGAAAC (R) CAAAGCTTGGCCCGACAT

SM30E SPU_004867 (F) CCAAGCCCAGTTCCAAGGA (R) GTTGGCGATTACCAGGTTGAC

RPL8 SPU_ 010692 (F) GCCATGGTCGGAGTTGTTG (R) TTGCGTTTGACCTTGTACTTGAA

Profilin SPU_ 020197 (F) CGCCAACTCCTTTATTTGTATTCC (R) TCTTTTTCCACTCTACAGCGATTG

Cyc7 SPU_ 008305 (F) AGTTTCCATCGTGTCATC (R) CTGTGATCCATTGGTGTT

ATub5 SPU_019990 (F) GGATGCTGCCAACAACTATGC (R) CGATCCAAGACTTGATCAATGAGT

PARP1 SPU_018184 (F2) CAAGCCTTTCAAGAAGCTCCAT (R2) GCCCCTGAGACAGGATTCC

HPRT SPU_002998 (F) CACACCATGCAGAGGTTACTCAA (R) TCCTTGGAGTCCTCTTCACTAATAGAC

PCNA SPU_023334 (F) GCATTCCAGACACAGAATATGCA (R) GGCTCAGATCTCGACAAATCCT

B tubulin2 SPU_000061 (F) CGAGAGGATCAACGTCTACTACAATG (R) CCATCGTACCTGGCTCCAGAT

GAPDH SPU_ 007155 (F) GCCTTGTCAATGGTTGTGAATACA

(R) GAATGAAGCCATCTGAGATTCCA

mActin SPU_006797 (F) GGTCAGGTCATCACTATTG

(R) GCTGTTGTATGTGGTCTC
